# Interaction of self-efficacy and sense of personal control on diabetes distress in older adults with type 2 diabetes: a cross-sectional study

**DOI:** 10.3389/fpubh.2026.1762971

**Published:** 2026-02-04

**Authors:** Zhaoxia Tian, Xia Ren, Quanyi Wang, Wenke Guo, Hongmei Li, Linping Shang

**Affiliations:** 1 Department of Nursing, Fenyang College of Shanxi Medical University, Fenyang, Shanxi Province, China; 2 Department of Nursing, Lvliang Hospital of Traditional Chinese Medicine, Lüliang, Shanxi Province, China; 3 Department of Internal Medicine, Fenyang Hospital of Shanxi Province, Fenyang, Shanxi Province, China; 4 Department of Nursing, The First Hospital of Shanxi Medical University, Taiyuan, Shanxi Province, China

**Keywords:** disease distress, elderly, personal mastery, self-efficacy, type 2 diabetes mellitus

## Abstract

**Background:**

Diabetes distress (DD) constitutes a significant barrier to effective diabetes management, impacting self-care behaviors and complication incidence rates. It directly influences quality of life and healthcare resource utilization among gerontal patients. Research on alleviating DD offers a novel perspective for developing personalized self-management plans in clinical practice.

**Methods:**

This cross-sectional study randomly selected 342 older adults with type 2 diabetes registered at community hospitals. Factors influencing disease distress were assessed using a demographic questionnaire, Diabetes Distress Scale (DDS), Personal Mastery Scale (PMS), General Self-Efficacy Scale (GSES), and Diabetes Self-Management Behavior Scale (DSMB-O). Structural equation modeling was employed for moderated mediation analysis.

**Results:**

Findings revealed that 59.06% of older adults with T2DM experienced distress. Independent risk factors for DDS included disease duration ≥10 years (*β* = 1.181), living alone (*β* = 1.592), low educational attainment (*β* = −1.639), low household income (*β* = 1.432), PMS score <20 (*β* = 0.828), and GSES score <30 (*β* = 0.887). Under the hypothetical model, the structural equation model revealed that the indirect path coefficients for self-efficacy and general sense of control via self-management on perceived illness distress were significant (−0.081, −0.066, *p* < 0.005). Concurrently, the chained path was also significant (−0.020, −0.009, *p* < 0.05).

**Conclusion:**

Factors influencing disease distress in older adults with T2DM are diverse. The complex interplay between personal mastery and self-efficacy offers new intervention strategies for clinically alleviating diabetes distress.

## Introduction

Given the lifelong nature of Type 2 diabetes mellitus (T2DM), patients face complex medical and disease-specific information (1), including managing chronic conditions, related symptoms, and complications. They also require high proficiency in sustained care activities throughout the disease course, such as adhering to medication regimens, regularly monitoring blood glucose levels, adjusting diets, following exercise plans, and implementing lifestyle changes. Research indicates that adults with type 2 diabetes exhibit significant deficiencies in self-management capabilities (2). Approximately 38% do not engage in daily exercise or foot care, 55% fail to follow dietary guidelines, and over one-third demonstrate poor adherence to diabetes medications. However, this persistent burden of disease management may trigger emotional responses such as anxiety and frustration in patients. Diabetes distress (DD) impacts patients’ perceptions of available support, emotional burden, and access to quality healthcare.

Research indicates that over half of people with diabetes experience emotional burden and stress related to their condition (3). Older patients face an even greater psychological burden, with approximately 40% of seniors experiencing more severe DD due to difficulties effectively managing their diabetes. This significantly impacts disease control, outcomes, and overall quality of life (4). Clinical data indicate that high levels of DD can elevate glycated hemoglobin by up to 1.2%. As DD-related complications and mortality rates rise, psychological interventions within treatment activities can no longer be overlooked. The severity of DD correlates significantly with multiple factors: personal factors include personality traits, coping mechanisms, and support networks; social factors encompass socioeconomic status, access to healthcare, and cultural influences (5). Management of DD depends on individual needs and intrinsic motivation (6).

Self-efficacy exhibits a bidirectional negative relationship with DD. Enhanced self-efficacy significantly reduces patients’ negative emotional responses, further promoting effective self-management of diabetes among older adults and improving their overall quality of life (7). Conversely, the emotional burden of DD consumes cognitive resources, diminishes patients’ confidence in executing self-management, and weakens self-efficacy. Research indicates that for every one-unit increase in distress, self-efficacy declines by 23% (8). Consequently, low self-efficacy has been identified as a risk factor for distress (9).

The sense of personal control, as a protective psychosocial resource, significantly influences the incidence of severe complications and the likelihood of hospitalization and premature mortality, reflecting an individual’s coping level with chronic illness (10). Particularly when poorly controlled type 2 diabetes progresses from oral medications to insulin therapy, patients often exhibit low engagement with the injection regimen, perceive themselves as having little control over T2D, and lack motivation for self-management due to perceived failure to achieve treatment goals (11). Conversely, patients with higher self-management competence can fully mobilize intrinsic psychological resources such as optimism and resilience to cope with psychological distress, thereby mitigating its negative impact.

Cognitive Social Theory (12) as a core theoretical framework for explaining the interaction between individual psychology and behavior, provides crucial theoretical support for understanding the relationship between self-efficacy, perceived personal control, and diabetes distress among gerontal patients with type 2 diabetes. This theory emphasizes the dynamic interplay between an individual’s cognitive processes (such as beliefs, expectations, and attributions), their social environment, and behavioral outcomes. Individuals are not passive recipients of environmental stimuli but actively regulate their behavior and emotional responses through perceptions of their own capabilities (self-efficacy), beliefs about controlling behavioral outcomes (personal control), and cognitive interpretations of their social environment. Within the diabetes management context, Cognitive Social Theory posits that patients’ cognitive appraisals of management tasks (e.g., ‘Do I possess the capacity to perform blood glucose monitoring?’;‘Can my actions effectively control my condition?’), feedback from social support networks including family and healthcare professionals, and accumulated experience in disease management collectively shape their self-efficacy and personal control. These factors subsequently influence the implementation of self-management behaviors and the emergence of diabetes distress.

High self-efficacy is associated with lower negative emotional responses in patients, and is linked to more effective self-management behaviors and higher overall quality of life. From a cognitive-social perspective, patients with high self-efficacy develop the cognitive belief that ‘they possess the capacity to manage diabetes challenges’ based on prior successful disease management experiences or positive feedback from healthcare professionals. This positive cognition encourages them to maintain proactive behavioral tendencies when confronting complex management tasks such as dietary control and regular exercise, thereby reducing anxiety and frustration arising from task difficulty. Conversely, the emotional burden of diabetes depletes cognitive resources, undermining patients’ confidence in executing self-management. This fosters negative cognitions of ‘inability to cope,’ thereby diminishing self-efficacy.

Personal sense of control, as the core manifestation of “beliefs about control” within cognitive social theory, constitutes a protective psychosocial resource. It exhibits significant correlations with the incidence of severe complications, likelihood of hospitalization, and premature mortality rates, reflecting an individual’s capacity to manage chronic disease. According to cognitive social theory, personal sense of control stems from an individual’s perception of the ‘behavior-outcome’ relationship: when patients perceive that their self-management behaviors (such as adhering to medication schedules and maintaining a healthy diet) can effectively control blood glucose levels and reduce complication risks, they develop positive control beliefs that their condition is manageable. Conversely, if they are unable to improve their condition through their own actions over the long term, or lack a clear understanding of disease progression, they develop a sense of loss of control. Particularly when poorly controlled type 2 diabetes progresses from oral medication to insulin therapy, patients often experience heightened negative cognition that ‘the condition is beyond their control’ due to unfamiliarity with the injection regimen and uncertainty about treatment efficacy. This frequently manifests as low adherence to the injection plan and diminished motivation for self-management. Conversely, patients with higher self-management capacity, grounded in the cognitive belief that their condition is controllable, can fully mobilize internal psychological resources (such as optimism and resilience) to cope with psychological distress, thereby mitigating its negative impact.

Research reveals that the essence of DD stems from an imbalance between disease demands and self-management capacity. From the perspective of cognitive social theory, the core of this imbalance lies in the mismatch between patients’ cognitive assessments and their disease management needs. When gerontal patients face complex disease management challenges, such as insulin regulation, dietary calculations, and complication monitoring, the absence of essential psychological resources (Self-efficacy and cognitive beliefs) may create a vicious cycle where the psychological burden of diabetes interacts with disease demands. This can negatively impact self-care behaviors and overall well-being (13).

Against this backdrop, exploring protective mechanisms for psychological behavior has emerged as a novel avenue for overcoming challenges. Guided by cognitive social theory, this study proposes a theory-driven hypothetical model to investigate the path relationships between self-efficacy, personal sense of control, self-management, and diabetic distress. Given the cross-sectional design of this research, the model cannot be validated by the data and serves solely to estimate path coefficients within the hypothetical framework.

*H*1: Self-efficacy significantly negatively predicts diabetes distress.

*H*2: Personal sense of control significantly negatively predicts diabetes distress.

*H*3: Self-efficacy significantly positively predicts self-management behaviors.

*H*4: Personal sense of control significantly positively predicts self-management behaviors.

*H*5: Self-management behaviors significantly negatively predict diabetes distress.

*H*6: Self-management behaviors mediate the relationship between self-efficacy and diabetes distress.

*H*7: Self-management behaviors mediate the relationship between personal control and diabetes distress.

*H*8: Self-efficacy and personal control indirectly influence diabetes distress via chained pathways (self-efficacy→personal control→self-management behaviors; personal control→self-efficacy→ self-management behaviors).

This model is grounded in the following literature: our selection of self-efficacy and personal sense of control as antecedents to diabetic distress is based on randomized controlled trials demonstrating that these factors predict changes in diabetic distress levels (14, 15). According to cognitive social theory, self-efficacy and personal control influence psychological adaptation precisely because they first enhance self-management behaviors; without modeling this pathway, the intermediate process would remain fragmented (16). Ji (17) research indicates that self-efficacy and personal sense of control may exhibit a dynamic, mutually beneficial chain relationship. Therefore, the model captures the conditional process whereby they empower behavioral improvements to alleviate diabetes distress (4). Nevertheless, this study cannot establish a causal relationship between self-efficacy, personal sense of control, and diabetes distress.

## Methods

### Study design

This study employs a cross-sectional design to explore factors influencing diabetes distress among gerontal T2DM patients and their interactions with self-efficacy and personal sense of control.

### Sample/participants

A total of 342 gerontal T2DM patients registered at a community hospital between December 2023 and December 2024 were selected (Total number of visitors: 480 Actual number of participants: 376 Participation rate: 78.3%). Inclusion criteria: (1) Diagnosis of T2DM; (2) Age ≥60 years; (3) Disease duration ≥6 months; (4) Clear consciousness and communication ability. Exclusion criteria: (1) Severe language impairment or cognitive dysfunction; (2) Concurrent critical illness or malignancy; (3) Other types of diabetes. According to the SEM sample size calculation method, the sample size should be 5 to 10 times the number of observed variables. This study has 48 observed variables. Considering a 20% non-response rate, the required sample size ranges from 288 to 576. The study obtained an effective sample size of 342 cases, which is sufficient to meet the model fitting requirements.

### Instruments

A combination of electronic record retrieval and face-to-face follow-up was employed to survey enrolled patients using the following four scales: (1) General Information Questionnaire, including gender, age, disease duration, marital status, living conditions, educational attainment, treatment modality, complications, and per capita monthly household income. (2) Diabetes Distress Scale (DDS): Comprising four dimensions—interpersonal distress, physician-related distress, routine-related distress, and emotional burden—with 17 items scored from 1 to 6. A mean total score of 3 or higher indicates clinical disease distress. Cronbach’s alpha coefficient: 0.967. (3) Personal Mastery Scale (PMS): 7 questions scored 1–5 points each, total range 7–35 points. Score ≥20 indicates good personal mastery; score <20 indicates poor personal mastery. Cronbach’s alpha coefficient: 0.932. (4) General Self-Efficacy Scale (GSES): Consists of 10 questions scored from 1 to 4 points each, yielding a total score range of 10 to 40 points. A score ≥30 indicates good self-efficacy, while a score <30 indicates low self-efficacy. Cronbach’s alpha coefficient: 0.949. (5) Diabetes Self-Management Behavior Scale (DSMB-O): Includes 7 dimensions: active exercise, healthy diet, medication adherence, blood glucose monitoring, managing hypoglycemia, positive coping, and reducing complication risks: 14 items. Total score range: 0–30 points. Higher scores indicate better diabetes self-management behavior. Cronbach’s alpha coefficient: 0.944.

### Data collection

All questionnaires were uniformly distributed and explained by two nurses. If patients struggled to complete the forms independently, the two nurses provided guidance to ensure the quality of all completed surveys, which were then collected, statistically compiled, and organized. This study distributed 376 questionnaires. After excluding 34 invalid questionnaires containing missing or erroneous entries, 342 valid questionnaires were recovered, yielding a valid response rate of 90.9%.

### Data analysis

Data were analyzed using SPSS 21.0 software. Count data were expressed as percentages (%), and chi-square tests were performed. Multivariate logistic regression analysis was used to examine factors influencing disease distress in gerontal T2DM patients. Statistical analysis of inferential mediating pathways in structural equation modeling under hypothetical frameworks.

## Results

### Univariate analysis of disease distress in older adults with type 2 diabetes

The mean disease distress score among older adults with type 2 diabetes was 60.56 ± 23.61 points. Univariate analysis revealed significant associations between disease distress and the following factors: female gender (χ^2^ = 22.66), longer disease duration (χ^2^ = 23.12), living alone (χ^2^ = 56.07), lower educational attainment (χ^2^ = 59.81), lower household income (χ^2^ = 47.82), higher PMS scores (χ^2^ = 19.35), and higher GSES scores (χ^2^ = 38.02), PMS scores (χ^2^ = 19.35), and GSES scores (χ^2^ = 38.02) were significantly associated with diabetes distress. These factors were therefore selected for inclusion in subsequent multivariate analysis models (Table 1).

**Table 1:** Univariate analysis of disease distress in older adults with type 2 diabetes.

Related factors		Number of cases	Distress		No distress		χ^2^	*p*
			*n* = 202		*n* = 140			
			Number of cases	Percentage	Number of cases	Percentage		
Gender	Male	167	77	46.11	90	53.89	22.66	0.000**
	Female	175	125	71.43	50	28.57		
Duration of illness	>10 years	94	36	38.30	58	61.70	23.121	0.000**
	<10 years	248	166	66.94	82	33.06		
Marital status	Married	228	128	56.14	100	43.86	2.419	0.120
	Divorced or unmarried	114	74	64.91	40	35.09		
Living arrangements	Living with family	191	79	41.36	112	58.64	56.073	0.000**
	Living alone	151	123	81.46	28	18.54		
Educational attainment	Elementary school and below	164	132	80.49	32	19.51	59.814	0.000**
	Junior high school and above	178	70	39.33	108	60.67		
Treatment method	Oral medication	222	130	58.56	92	41.44	0.073	0.964
	Insulin injection	78	47	60.26	31	39.74		
	Oral + insulin injection	42	25	59.52	17	40.48		
Complications	None	27	18	66.67	9	33.33	1.972	0.373
	1 type	75	48	64.00	27	36.00		
	2 or more	240	136	56.67	104	43.33		
Per capita household income	≥5,000 yuan	87	24	27.59	63	72.41	47.818	0.000**
	Less than 5,000 yuan	255	178	69.80	77	30.20		
PMS score	≥20 points	171	81	47.37	90	52.63	19.349	0.000**
	<20 points	171	121	70.76	50	29.24		
GSES score	≥30 points	138	54	39.13	84	60.87	38.022	0.000**
	<30 points	204	148	72.55	56	27.45		

*p < 0.05; **p < 0.001.

### Multivariate logistic regression analysis of disease distress in older adults with type 2 diabetes

Regression analysis revealed that short disease duration (*β* = 1.181), living alone (*β* = 1.592), low educational attainment (*β* = −1.639), low household income (*β* = 1.432), low PMS score (*β* = 0.828), and low GSES score (*β* = 0.887) were independent predictors of suffering in gerontal diabetic patients (Table 2).

**Table 2:** Multivariate logistic regression analysis of disease distress in older adults with type 2 diabetes.

Variable	*β*	SE	Wald *χ^2^*	*p*	Odds ratio	95% CI
Gender	0.513	0.301	2.909	0.088	1.670	0.926–3.010
Duration of illness	1.181	0.329	12.869	0.000	3.258	1.709–6.212
Housing Conditions	1.592	0.314	25.650	0.000	4.912	2.653–9.094
Educational attainment	−1.639	0.303	29.293	0.000	0.194	0.107–0.352
Per capita household income	1.432	0.345	17.200	0.000	4.185	2.128–8.233
PMS score	0.828	0.302	7.514	0.006	2.290	1.266–4.140
GSES score	0.887	0.307	8.323	0.004	2.428	1.329–4.435

### Structural equation modeling of disease-related distress in gerontal patients with type 2 diabetes mellitus

**Figure 1:**
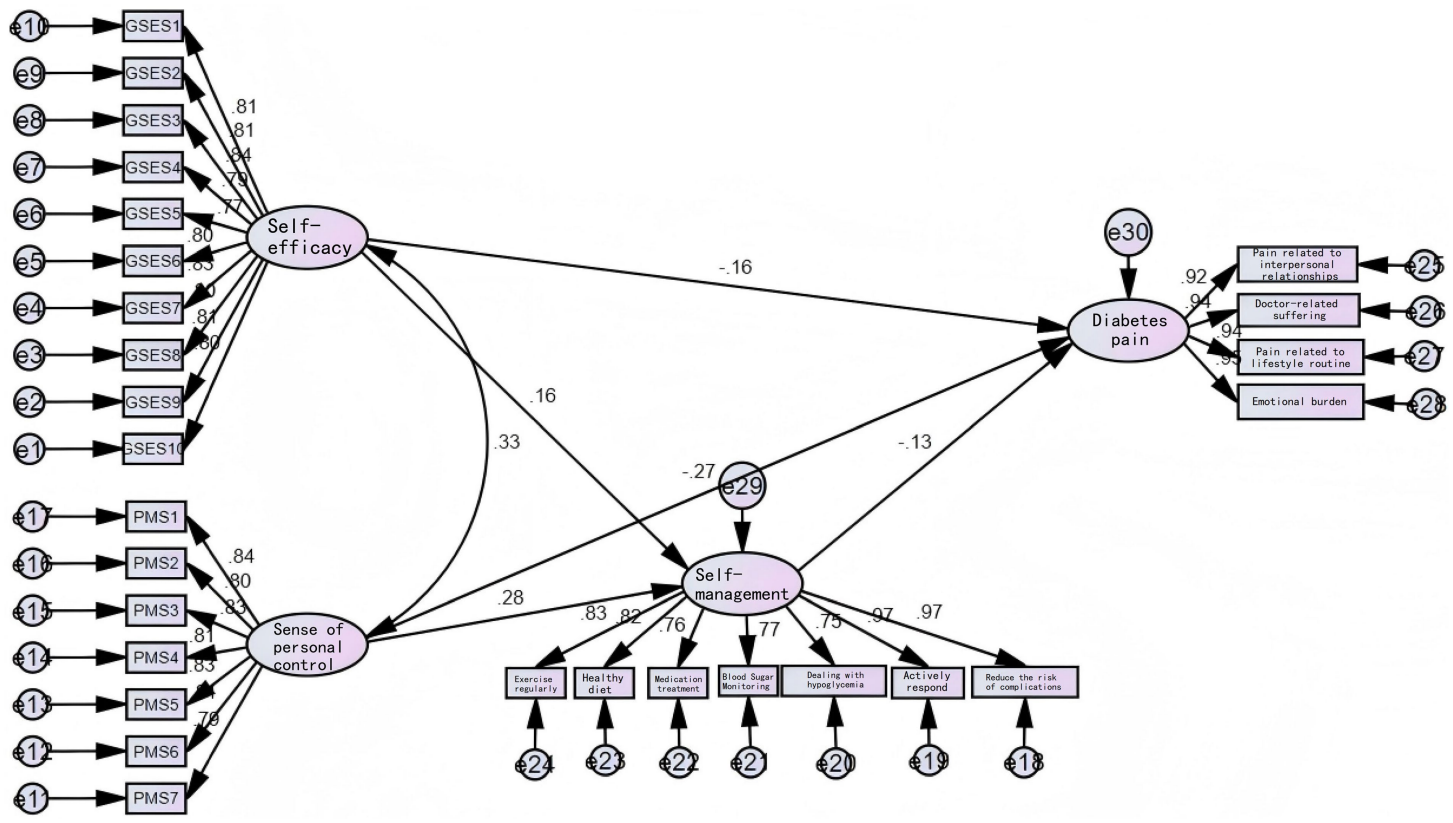
Structural equation model of disease distress in gerontal type 2 diabetes patients. The pathways depicted in the figure should be regarded as exploratory findings rather than validation of causal mechanisms.

In the structural equation model fit assessment, the absolute fit index χ^2^/df stands at 2.285, indicating a balanced equilibrium between model complexity and fit (Figure 1). The RMSEA value of 0.061 suggests the model’s approximation error remains within acceptable limits, while the GFI of 0.844 demonstrates overall satisfactory data fit. All relative fit indices achieved excellent levels in empirical testing. The IFI, TLI, and CFI (0.951, 0.945, 0.950) confirm that the model’s relative fit and explanatory power are both excellent, effectively supporting the testing of research hypotheses. The actual values of PNFI and PCFI are also significantly above 0.5, indicating that the model maintains high fit while possessing good parsimony. This demonstrates that the structural equation model constructed in this test exhibits good model fit (Table 3).

**Table 3:** Model fit tests.

Fit measure	Reference standard	Observed value
χ^2^/df	1–3: Excellent, 3–5: Good	2.285
RMSEA	<0.05: Excellent, <0.08: Good	0.061
GFI	>0.9: Excellent, >0.8: Good	0.844
AGFI	>0.9: Excellent, >0.8: Good	0.816
IFI	>0.9: Excellent, >0.8: Good	0.951
TLI	>0.9: Excellent, >0.8: Good	0.945
CFI	>0.9: Excellent, >0.8: Good	0.950
PCFI	>0.5	0.865
PNFI	>0.5	0.833

The path relationship test results of the structural equation model indicate that all hypothesized paths proposed in this study reached the level of statistical significance (*p* < 0.05). Specifically, significant negative relationships were found between self-efficacy (−0.16), general sense of control (−0.27), self-management (−0.13), and distress among gerontal diabetic patients. These factors can alleviate the perception of disease distress (Table 4).

**Table 4:** Path relationship tests in structural equation modeling.

Path relationship			Estimate	S. E.	C. R.	*p*
Self-management	←	Self-Efficacy	0.158	0.019	2.788	0.005
Self-management	←	General Sense of Control	0.283	0.014	4.912	***
Diabetes pain	←	Self-Efficacy	−0.161	0.086	−2.869	0.004
Diabetes distress	←	Self-Management	−0.134	0.258	−2.442	0.015
Diabetes suffering	←	General Sense of Control	−0.268	0.066	−4.545	***
GSES10	←	Self-Efficacy	0.796			
GSES9	←	Self-Efficacy	0.812	0.059	17.107	***
GSES8	←	Self-Efficacy	0.801	0.062	16.792	***
GSES7	←	Self-Efficacy	0.831	0.061	17.638	***
GSES6	←	Self-Efficacy	0.797	0.061	16.679	***
GSES5	←	Self-Efficacy	0.774	0.062	16.021	***
GSES4	←	Self-Efficacy	0.795	0.062	16.608	***
GSES3	←	Self-efficacy	0.840	0.059	17.917	***
GSES2	←	Self-Efficacy	0.810	0.061	17.045	***
GSES1	←	Self-Efficacy	0.806	0.060	16.925	***
PMS7	←	General sense of control	0.787	0.054	17.300	***
PMS6	←	General sense of control	0.806	0.055	17.934	***
PMS5	←	General sense of control	0.829	0.051	18.745	***
PMS4	←	General sense of control	0.806	0.054	17.936	***
PMS3	←	General sense of control	0.835	0.053	18.946	***
PMS2	←	General sense of control	0.799	0.054	17.688	***
PMS1	←	General sense of control	0.838			
Reduced risk of complications	←	Self-Management	0.973			
Active coping	←	Self-Management	0.972	0.082	51.839	***
Managing hypoglycemia	←	Self-Management	0.755	0.144	20.201	***
Blood glucose monitoring	←	Self-Management	0.768	0.138	21.015	***
Pharmacotherapy	←	Self-Management	0.756	0.151	20.273	***
Healthy eating	←	Self-Management	0.821	0.115	24.784	***
Active exercise	←	Self-Management	0.830	0.116	25.552	***
Interpersonal relationship-related distress	←	Diabetes-related suffering	0.922			
Doctor-related suffering	←	Diabetes-Related Suffering	0.941	0.031	32.478	***
Pain related to daily routine	←	Diabetes-related suffering	0.938	0.031	32.091	***
Emotional burden	←	Diabetes Suffering	0.948	0.031	33.390	***

***indicates *p* < 0.001.

This study employed a bootstrapping method with 5,000 iterations to calculate 95% confidence intervals, thereby further validating the mediating effects. Results indicate that the indirect effects of self-management were significant across all path relationships. Self-efficacy and general sense of control can directly influence diabetes distress and also exert indirect effects through the mediating variable of self-management (Table 5).

**Table 5:** Mediation effect results.

Path relationship	Effect	Boot SE	BootLLCI	BootULCI	z	*p*
Self-efficacy → Self-management → Diabetes distress	−0.081	0.020	−0.096	−0.022	−4.150	0.000
General sense of control → Self-management → Diabetes distress	−0.066	0.022	−0.104	−0.020	−3.037	0.002

BootLLCI denotes the lower bound of the 95% Bootstrap confidence interval, BootULCI denotes the upper bound of the 95% Bootstrap confidence interval. Bootstrap type: Percentile Bootstrap method.

This study employed the Bootstrap method to construct a chained mediation model, conducting 5,000 repeated samples and calculating 95% confidence intervals. General sense of control indirectly influences diabetes distress through self-efficacy (Effect = −0.057), self-management (Effect = −0.048), and the dual chained path “self-efficacy→self-management” (Effect = −0.009). Self-efficacy also indirectly influences diabetes distress through general sense of control (Effect = −0.118), self-management (Effect = −0.038), and the chained path “general sense of control→ self-management” (Effect = −0.020). The confidence intervals for all three paths did not include zero, indicating significant mediating effects (Table 6).

**Table 6:** Chain mediation effect analysis.

Chain mediation	Effect	Boot SE	BootLLCI	BootULCI	z	*p*
General sense of control → Self-efficacy → Diabetes distress	−0.057	0.021	−0.094	−0.014	−2.760	0.006
General sense of control → Self-management → Diabetes distress	−0.048	0.018	−0.082	−0.011	−2.611	0.009
General sense of control → Self-efficacy → Self-management → Diabetes distress	−0.009	0.004	−0.018	−0.001	−2.112	0.035
Self-efficacy → General sense of control → Diabetes distress	−0.118	0.024	−0.130	−0.037	−4.922	0.000
Self-efficacy → Self-management → Diabetes distress	−0.038	0.015	−0.061	−0.003	−2.527	0.012
Self-efficacy → General sense of control → Self-management → Diabetes distress	−0.020	0.005	−0.023	−0.004	−4.064	0.000

BootLLCI denotes the lower bound of the 95% Bootstrap confidence interval, BootULCI denotes the upper bound of the 95% Bootstrap confidence interval, Bootstrap type: percentile Bootstrap method.

## Discussion

### Current status of disease burden in older adults with type 2 diabetes

The INTERPRET-DD and DAWN studies demonstrated that diabetes distress warrants global attention (18). Studies indicate varying prevalence rates across countries and regions. A meta-analysis of DD prevalence among US T2D individuals reported rates ranging from 19 to 79.5% (19). A cross-sectional study of South Asian Canadians revealed a high prevalence of 52.5% (20). Furthermore, DD tends to worsen over time (16, 21), and there is a significant gap in meeting patients’ mental health care needs (22). Personalized interventions are particularly crucial for alleviating distress at different stages of the disease and addressing diverse psychological needs. Research indicates that order participants receiving personalized interventions experience greater reductions in distress than younger participants (23), demonstrating that focusing on reducing distress among order diabetes patients holds significant clinical importance and yields notable outcomes.

### Factors influencing disease distress in older adults with type 2 diabetes

This study reveals that in sociodemographic surveys, living alone, long disease duration, low educational attainment, and low household income are risk factors for DD in older adults with T2DM. Patients living alone experienced higher distress levels than those with family support, consistent with findings by Li Wen (24) and others. For Chinese individuals with strong family-centered values, losing this vital support channel prevents them from gaining emotional support and coping strategies through family functions. This leaves patients feeling helpless and exhausted when confronting complex management scenarios alone. Studies on the impact of disease duration on DD remain inconclusive. This research found that patients with longer disease duration experience increased fear of complications and stigmatization, coupled with the burden of prolonged self-care, leading to management neglect and a sense of loss of control as the disease progresses. Luzuriaga et al. (25) also found that patients with long-standing diabetes may experience greater distress. Conversely, some studies suggest that patients with longer disease duration have attained self-care levels through improved disease cognition and management skills, established blood glucose-maintaining habits, developed complication monitoring abilities, and achieved medication adherence, resulting in relatively lower distress levels (26). This may relate to the continuity of healthcare access and individual knowledge acquisition among the selected study subjects. Educational attainment is an independent risk factor for diabetes distress. Lower educational levels increase patients’ disease distress, consistent with findings by Li et al. (27). This may stem from patients’ greater reliance on non-professional sources (e.g., friends’ or relatives’ experiences), difficulties in understanding self-management knowledge (e.g., diet, exercise, medication), and lack of effective emotional regulation strategies. Lower household income correlates with higher disease distress. As a lifelong metabolic disorder, diabetes requires long-term medication and continuous monitoring. Some patients necessitate high-cost treatments like insulin or rapid-acting insulin. However, prolonged, substantial treatment expenses often trap low-income patients in a “cost-compliance” dilemma, leading to reduced or discontinued medication. This undermines glycemic control and exacerbates psychological distress. Notably, multivariate regression analysis revealed no direct association between gender and diabetes distress, consistent with Chen Y-C’s findings (23). However, Tesfa research suggests that (24, 28, 29). Women are more prone to diabetes-related issues, possibly due to differences in emotional expression between genders or variations in physiological hormones, which may lead women to exhibit higher levels of anxiety. The impact of gender on distress among gerontal type 2 diabetes patients remains unclear, and further well-powered studies are warranted to clarify its effect.

### Interaction between self-efficacy, general sense of control, and disease distress

This study’s path analysis aligns with theoretical assumptions. Self-efficacy and personal sense of control may independently influence diabetes distress, while also exerting indirect effects through the mediating variable of self-management. However, owing to the cross-sectional design, only path strengths within the hypothesized model can be assessed. Consequently, the mediation analysis in this study should be regarded as exploratory rather than confirmatory.

Strong self-efficacy and a greater sense of personal control are both significantly associated with lower levels of distress among individuals with diabetes. Enhanced self-efficacy markedly diminishes negative emotional responses in patients, consistent with findings from Niko Verdecias et al. (30). High self-efficacy as an individual’s belief, judgment, or subjective perception regarding diabetes management, typically mobilizes positive emotions and motivates patients to proactively seek medical assistance. Furthermore, self-efficacy serves as a crucial predictor for improved diabetes self-management (31). It further catalyzes the transition toward self-management behaviors, prompting patients to adopt effective coping strategies, adhere to treatment plans, and maintain resilience to alleviate distress, thereby enhancing the well-being of both patients and their support networks (32). The theoretical pathway of this study posits that personal control serves as a protective factor in diabetes management, enabling better control of chronic conditions requiring self-management. As self-management demands grow more complex and burdensome, control appears increasingly effective (11). Enhancing individuals’ perceived control over their disease, particularly when medication regimens require adjustment, may foster greater insight to improve patient engagement and adherence. Individuals with higher levels of perceived control are more likely to engage in better self-management, develop health-promoting beliefs, participate in community health education, and even learn to use smart devices to assist in management.

The path hypothesis posits that self-efficacy and general sense of control exhibit both synergistic effects and partial mediating chain effects. Synergy may arise from their shared psychological mechanisms, both mobilizing patients’ positive attitudes and beliefs toward disease management. This fosters self-management behaviors, enhancing confidence and capability to execute health actions such as personalized consultations, dietary modifications, structured physical exercise, and stress management techniques (33). This positive belief is significantly associated with alleviating negative emotions such as anxiety and depression, as well as psychological distress (34), also encourages patients to actively participate in diabetes treatment decisions, seek medical resources and social support, and improve quality of life. Partial mediation occurs by enhancing patients’ perceived control over their disease, thereby boosting self-efficacy, reducing feelings of helplessness in treatment, significantly increasing confidence in successfully establishing self-management support systems, and indirectly alleviating distress through positive behavioral feedback such as improved HbA1c levels. Patients’ confidence in self-management can significantly enhance their subjective sense of control over symptoms by increasing their proactive involvement in disease management. This, in turn, drives the establishment and implementation of management behaviors, such as independently seeking community education, thereby alleviating the distress associated with diabetes.

In summary, the factors contributing to the suffering associated with diabetes are multifaceted, and path estimates within the hypothetical model reveal partial mediating effects. When developing clinical treatment and lifelong management plans, psychological interventions should be integrated into care protocols. For instance, cognitive behavioral therapy and other interventions can facilitate the construction of multidimensional self-management systems, enhance patients’ resilience against the disease, maintain stable internal bodily environments, and reduce the risk of long-term complications. Concurrently, this approach improves the efficiency of healthcare resource utilization and alleviates the societal burden of medical care.

## Conclusion

This study demonstrates that self-efficacy, general sense of control, and demographic factors (longer disease duration, living alone, lower education level, lower household income) predict disease distress in gerontal T2MD patients. Within the theoretical framework, self-efficacy and general sense of control may be associated with perceived illness distress either independently or indirectly via the mediating pathway of enhanced self-management. Furthermore, the chain-like interactive relationship between self-efficacy and sense of control offers a novel perspective for elucidating the underlying mechanisms influencing illness distress. This provides precise targets for designing phased intervention programs, stimulating intrinsic motivation, and alleviating emotional burdens stemming from fear of complications and cumbersome management.

## Limitations

Due to the selection of an order population, patients had already established partial disease management patterns. This study could not clarify whether self-efficacy and personal sense of control could still yield positive outcomes when diabetes distress scores were excessively high. Future research may explore these variables to further refine the interplay among the three factors. Furthermore, as this study employs a cross-sectional design, the mediating pathway should be regarded as an exploratory finding. Its causal nature requires further validation through longitudinal or experimental designs in future research.

## Data Availability

The raw data supporting the conclusions of this article will be made available by the authors, without undue reservation.

## References

[1] Mitchell SE Kallen MA Troost JP de la Cruz BA Bragg A Martin-Howard J . Four new patient-reported outcome measures examining Health-seeking behavior in persons with type 2 diabetes mellitus (REDD-CAT): Instrument development study. JMIR Diabetes. (2024) 9:e63434. doi: 10.2196/63434, 39576685 PMC11624447

[2] Nicolucci A Kovacs Burns K Holt RI. Diabetes attitudes, wishes and needs second study (DAWN2™): Cross-national benchmarking of diabetes-related psychosocial outcomes for people with diabetes. Diabet Med. (2013) 30:767–77. doi: 10.1111/dme.12245 23711019

[3] Poole L Hackett R. Diabetes distress: the psychological burden of living with diabetes. Lancet Diabetes Endocrinol. (2024) 12:439–41. doi: 10.1016/S2213-8587(24)00126-8 38824928

[4] Rubio-Almanza M Cámara-Gómez R Merino-Torres JFJE. Obesidad y diabetes mellitus tipo 2. Endocrinol Diabetes Nutr. (2019) 66:140–9. doi: 10.1016/j.endinu.2018.08.003 30337188

[5] Sendekie AK Limenh LW Bizuneh GK Kasahun AE Wondm SA Tamene FB . Psychological distress and its impact on glycemic control in patients with diabetes, northwest Ethiopia. Front Med (Lausanne). (2025) 12:1488023. doi: 10.3389/fmed.2025.1488023 40206466 PMC11979121

[6] Chandran SR Keat GSK Salim NNBM. Beyond glycaemia: socioeconomic factors and diabetes distress are associated with Health-related quality of life in people with type 2. Diabetes. (2025) 40:72. doi: 10.15605/jafes.040.01.19 PMC12097981 40416482

[7] Gómez-Velasco DV Almeda-Valdes P Martagón AJ Galán-Ramírez GA Aguilar Salinas CA. Empowerment of patients with type 2 diabetes: Current perspectives. DMSO. (2019) 12:1311–21. doi: 10.2147/DMSO.S174910 PMC6689555 31496769

[8] Huang Y-C Zuniga J Garcia AJE. Illness perceptions as a mediator between emotional distress and management self-efficacy among Chinese Americans with type 2 diabetes. Ethn Health. (2022) 27:672–86. doi: 10.1080/13557858.2020.1817339 32894684

[9] Zhou C Maitland E Nicholas S Tian X Liu RJIH. Medical alliances and diabetes-related distress in China: Role of self-efficacy as a partial mediator. Int Health. (2025) 17:968–77. doi: 10.1093/inthealth/ihaf040 40237098 PMC12585574

[10] Care ADAJD. 5. Facilitating behavior change and well-being to improve Health outcomes:standards of medical Care in Diabetes—2020. Diabetes Reviews. (2020) 43:S48–65. doi: 10.2337/dc20-S005 31862748

[11] McGuigan K Hill A Coates V O’Kane M Thompson DR Ski CF . Moderating the relationship between diabetes distress and mastery. Psychol Health Med. (2022) 27:838–47. doi: 10.1080/13548506.2021.1894343 33641545

[12] Miller SM Shoda Y Hurley K. Applying cognitive-social theory to health-protective behavior: breast self-examination in cancer screening. Psychol Bull. (1996) 119:70–94. 8559860 10.1037/0033-2909.119.1.70

[13] Fisher L Polonsky WH Hessler DJDM. Addressing diabetes distress in clinical care. Innovative care. (2019) 36:803–12. doi: 10.1111/dme.13967 30985025

[14] Wu SFV Lee MC Liang SY Lu YY Wang TJ Tung HH. Effectiveness of a self‐efficacy program for persons with diabetes. Plant Species Biol. (2011) 13:335–43. doi: 10.1111/j.1442-2018.2011.00625.x 21812879

[15] Wong AK Stewart AG Furler J. Development and validation of the Diabetes Management Orientation Scale (DMOS). Diabetes Res Clin Pract. (2009) 86:24–30. doi: 10.1016/j.diabres.2009.07.005 19671482

[16] Hadj-Abo A Enge S Rose J Kunte H Fleischhauer M. Individual differences in impulsivity and need for cognition as potential risk or resilience factors of diabetes self-management and glycemic control. PLoS One. (2020) 15:e0227995. doi: 10.1371/journal.pone.0227995 31995586 PMC6988919

[17] Ji M Ren D Dunbar-Jacob J Gary-Webb TL JAJNR E. Self-Management Behaviors, Glycemic Control, and Metabolic Syndrome in Type 2 Diabetes. Nurs Res. (2020) 69:E9–E17. doi: 10.1097/NNR.0000000000000401 32108739

[18] Qureshi Z Ali M. Diabetic Neuropathy Pain Management. Curr Diabetes Rev. (2021) 17:57–69. doi: 10.2174/1573399816666201103142521 33143631

[19] Perrin N Davies MJ Robertson N Snoek F Khunti KJDM. The prevalence of diabetes‐specific emotional distress in people with Type 2 diabetes. Innovative care. (2017) 34:1508–20. doi: 10.1111/dme.13448 28799294

[20] Sidhu R Tang T. Diabetes Distress and Depression in South Asian Canadians with Type 2 Diabetes. Can J Diabetes. (2017) 41:69–72. doi: 10.1016/j.jcjd.2016.07.008 27745846

[21] Fisher L Mullan JT Arean P Glasgow RE Hessler D Masharani U. Diabetes Distress but Not Clinical Depression or Depressive Symptoms Is Associated With Glycemic Control in Both Cross-Sectional and Longitudinal Analyses. Diabetes Rev. (2010) 33:23–8. doi: 10.2337/dc09-1238 PMC2797978 19837786

[22] DeVries C Rodríguez-Putnam A Ewen A Flores B Choudhary P Spring E . Protocol for the diabetes, distress and disparities (3D) study. BMJ Open. (2025) 15:e088082. doi: 10.1136/bmjopen-2024-088082 PMC11987096 40216429

[23] Swathi N Mohammed A Umar M Muhammad S Iqbal J. Evaluating the Impact of Individualized Interventions on Diabetes Distress and Glycemic Outcomes. Peer E med. (2025) 17:e80890. doi: 10.7759/cureus.80890 PMC12009104 40255758

[24] Chesla C. A. Fisher L Mullan J. T. Skaff M. M. Gardiner P Chun K . Family and disease management in African-American patients with type 2 diabetes. Diabetes care. (2004) 27, 2850–2855. doi: 10.2337/diacare.27.12.2850 15562196

[25] Luzuriaga M Leite R Ahmed H Saab PG. Complexity of antidiabetic medication regimen is associated with increased diabetes-related distress in persons with type 2 diabetes mellitus. BMJ Open Diabetes Res Care. (2021) 9:e002348. doi: 10.1136/bmjdrc-2021-002348 PMC8487180 34598934

[26] Liepinsh E Vilskersts R Zvejniece L Svalbe B Skapare E Kuka J . Protective effects of mildronate in an experimental model of type 2 diabetes in Goto‐Kakizaki rats. Br J Pharmacol. (2009) 157:1549–56. doi: 10.1111/j.1476-5381.2009.00319.x 19594753 PMC2765322

[27] Li C Wang W Ji Q Ran X Kuang H Yu X . Prevalence of painful diabetic peripheral neuropathy in type 2 diabetes mellitus and diabetic peripheral neuropathy. Diabetes Res Clin Pract. (2023) 198:110602. doi: 10.1016/j.diabres.2023.110602 36871876

[28] Habtewold TD Alemu SM Haile Y. Sociodemographic, clinical, and psychosocial factors associated with depression among type 2 diabetic outpatients in Black Lion General Specialized Hospital, Addis Ababa, Ethiopia. Psychiatry. (2016) 16:103. doi: 10.1186/s12888-016-0809-6 27083154 PMC4833927

[29] Dhamala E Christensen E Hanson JL. Neuroanatomy Reflects Individual Variability in Impulsivity in Youth. Res Square. (2025) 23:650222. doi: 10.21203/rs.3.rs-6520460/v1 PMC13364653 41876706

[30] Verdecias N McQueen A Von Nordheim DA. Diabetes distress in a Medicaid sample: the role of psychosocial and health-related factors. J Diabetes Complicat. (2023) 37:108495. doi: 10.1016/j.jdiacomp.2023.108495 PMC10330688 37156052

[31] Kerari AJD. Contribution of disease-specific distress, social support, and self-efficacy to diabetes self-management Behaviors in Saudi adults: a path analysis. Diabetes Metab Syndr Obes. (2024) 17:3991–4001. doi: 10.2147/DMSO.S479395 39492966 PMC11531232

[32] Gonzalez J. S. Peyrot M. McCarl L. A. Collins E. M. Serpa L Mimiaga M. J. . Depression and diabetes treatment nonadherence: a meta-analysis. Diabetes care. (2008). 31, 2398–2403. doi: 10.2337/dc08-1341 19033420 PMC2584202

[33] Lindekilde N Scheuer SH Rutters F Knudsen L Lasgaard M Rubin KH . Prevalence of type 2 diabetes in psychiatric disorders. Diabetologia. (2022) 65:440–56. doi: 10.1007/s00125-021-05609-x 34841451

[34] ElSayed NA Aleppo G Bannuru RR. 2. Diagnosis and classification of diabetes: Standards of care in diabetes—2024. Diabetes Care. (2024) 47:S20–42. doi: 10.2337/dc24-S002 38078589 PMC10725812

